# Co-developing climate services for public health: Stakeholder needs and perceptions for the prevention and control of *Aedes*-transmitted diseases in the Caribbean

**DOI:** 10.1371/journal.pntd.0007772

**Published:** 2019-10-28

**Authors:** Anna M. Stewart-Ibarra, Moory Romero, Avery Q. J. Hinds, Rachel Lowe, Roché Mahon, Cedric J. Van Meerbeeck, Leslie Rollock, Marquita Gittens-St. Hilaire, Sylvester St. Ville, Sadie J. Ryan, Adrian R. Trotman, Mercy J. Borbor-Cordova

**Affiliations:** 1 Institute for Global Health and Translational Science, State University of New York (SUNY) Upstate Medical University, Syracuse, New York, United States of America; 2 Department of Medicine and Department of Public Health and Preventative Medicine, SUNY Upstate Medical University, Syracuse, New York, United States of America; 3 InterAmerican Institute for Global Change Research (IAI), Montevideo, Department of Montevideo, Uruguay; 4 Department of Environmental Studies, SUNY College of Environmental Sciences and Forestry, Syracuse, New York, United States of America; 5 Caribbean Public Health Agency, Port of Spain, Trinidad & Tobago; 6 Centre on Climate Change and Planetary Health, London School of Hygiene & Tropical Medicine, London, United Kingdom; 7 Centre for Mathematical Modelling of Infectious Diseases, London School of Hygiene & Tropical Medicine, London, United Kingdom; 8 Barcelona Institute for Global Health (ISGlobal), Barcelona, Spain; 9 The Caribbean Institute for Meteorology and Hydrology, St. James, Barbados; 10 Ministry of Health and Wellness, St. Michael, Barbados; 11 Faculty of Medical Sciences, University of the West Indies at Cave Hill, Bridgetown, St. Michael, Barbados; 12 Best-dos Santos Public Health Laboratory, Ministry of Health, St. Michael, Barbados; 13 Environmental Health Division, Ministry of Health and Environment, Roseau, Commonwealth of Dominica; 14 Quantitative Disease Ecology and Conservation Lab Group, Department of Geography and Emerging Pathogens Institute, University of Florida, Gainesville, Florida, United States of America; 15 School of Life Sciences, University of KwaZulu-Natal, Durban, South Africa; 16 Facultad de Ingeniería Marítima y Ciencias del Mar, Escuela Superior Politécnica del Litoral (ESPOL), Guayaquil, Ecuador; Institute for Disease Modeling, UNITED STATES

## Abstract

**Background:**

Small island developing states (SIDS) in the Caribbean region are challenged with managing the health outcomes of a changing climate. Health and climate sectors have partnered to co-develop climate services to improve the management of emerging arboviral diseases such as dengue fever, for example, through the development of climate-driven early warning systems. The objective of this study was to identify health and climate stakeholder perceptions and needs in the Caribbean, with respect to the development of climate services for arboviruses.

**Methods:**

Stakeholders included public decision makers and practitioners from the climate and health sectors at the regional (Caribbean) level and from the countries of Dominica and Barbados. From April to June 2017, we conducted interviews (n = 41), surveys (n = 32), and national workshops with stakeholders. Survey responses were tabulated, and audio recordings were transcribed and analyzed using qualitative coding to identify responses by research topic, country/region, and sector.

**Results:**

Health practitioners indicated that their jurisdiction is currently experiencing an increased risk of arboviral diseases associated with climate variability, and most anticipated that this risk will increase in the future. National health sectors reported financial limitations and a lack of technical expertise in geographic information systems (GIS), statistics, and modeling, which constrained their ability to implement climate services for arboviruses. National climate sectors were constrained by a lack of personnel. Stakeholders highlighted the need to strengthen partnerships with the private sector, academia, and civil society. They identified a gap in local research on climate-arbovirus linkages, which constrained the ability of the health sector to make informed decisions. Strategies to strengthen the climate-health partnership included a top-down approach by engaging senior leadership, multi-lateral collaboration agreements, national committees on climate and health, and shared spaces of dialogue. Mechanisms for mainstreaming climate services for health operations to control arboviruses included climatic-health bulletins and an online GIS platform that would allow for regional data sharing and the generation of spatiotemporal epidemic forecasts. Stakeholders identified a 3-month forecast of arboviral illness as the optimal time frame for an epidemic forecast.

**Conclusions:**

These findings support the creation of interdisciplinary and intersectoral ‘communities of practice’ and the co-design of climate services for the Caribbean public health sector. By fostering the effective use of climate information within health policy, research and practice, nations will have greater capacity to adapt to a changing climate.

## Author summary

Small island nations in the Caribbean are highly vulnerable to climate change, whose effects include increased frequency and severity of droughts and increased intensity of tropical storms and hurricanes. Extreme weather and climate events affect directly or indirectly most dimensions of human well being, including mental and physical health, food, housing, freshwater, and livelihoods. Climate-driven early warning systems (EWS) to predict epidemics of dengue fever and other mosquito-borne diseases can help nations to adapt to changing climate conditions. In this study we assessed climate and health sector stakeholder perceptions and needs to inform the development of a dengue EWS. Stakeholders identified capacity limitations (financial resources, trained personnel), and the need for local research on climate-arbovirus linkages to inform decision makers. They identified six key strategies for strengthening the partnership between the climate-health sectors, and they assessed the viability of public health actions that could be take in response to short (2 week), medium (3 month) and long-term (1 year) forecasts of dengue fever epidemics. The results of this study contribute to Caribbean efforts to develop climate services for health, a key adaptation strategy to reduce the health impacts of climate change.

## Introduction

Small island developing states (SIDS) in the Caribbean region are highly susceptible to the health impacts of climate variability and long-term changes in climate [1,2]. Impacts include increased risk of communicable diseases, such as mosquito-borne arboviruses, and noncommunicable diseases, such as cardiovascular complications associated with heat stress. SIDS face similar challenges—small populations who are repeatedly exposed to extreme weather and climate events (e.g., droughts and tropical storms), limited global political power, reliance on imported goods, and difficulty preventing and responding to disasters due to resource constraints [1,3,4]. Caribbean SIDS will likely experience more extreme hydrometeorological events in the future due to climate change, thereby increasing the morbidity and mortality of climate-sensitive health events and increasing the associated social and economic costs.

Dengue fever, chikungunya and Zika fever are arboviral diseases transmitted by the *Aedes aegypti* and *Aedes albopictus* mosquitoes. Theses diseases among the top public health concerns in the Caribbean region [5,6]. In 2019, the Caribbean Public Health Agency (CARPHA) issued an advisory for the possibility of a severe dengue epidemic [7], given a rise in dengue activity in Latin America, the increasing burden of arboviruses over the last number of years, and the large gap in time since the last dengue epidemic in the Caribbean region, which occurred in 2009. The Caribbean is the region of the Americas with the highest incidence of dengue [8]. Vector control is the main public health intervention to prevent and control disease outbreaks through insecticide application, elimination of larval habitat sites, public education and community mobilization [9]. Despite these efforts, the annual number of dengue cases in the region increased from an estimated 136,000 to 811,000 cases between 1990 and 2013, with case estimates adjusted to account for underreporting [8]. New tools and management strategies are urgently needed to increase the capacity of the public health sector to prevent and respond to arboviral disease outbreaks.

Changes in local climate can influence mosquito vector physiology and population dynamics, thereby affecting disease transmission. Warmer ambient temperatures increase the probability of arbovirus transmission by *Ae*. *aegypti*, with optimum transmission at 28.5°C; however, transmission is improbable in extreme heat (>34°C) [10]. Both excess rainfall and drought conditions can potentially increase mosquito densities, depending on the characteristics of the built environment and anthropogenic water storage [11,12]. In the Caribbean region, studies have documented the effects of climate on dengue and *Ae*. *aegypti* in Barbados [11,13–16], Cuba [17], Puerto Rico [18–20], Jamaica [16,21], Trinidad and Tobago [13,16], and Guadeloupe [22].

Given the linkages between arboviruses, vectors, and climate, the World Health Organization (WHO) and experts in the Caribbean have recommended developing climate-driven early warning systems (EWS) and models to forecast arbovirus outbreaks [23,24]. These tools are known as *climate services*—tailored products for a specific sector that allow decision makers and practitioners to manage the risks posed by climate variability and climate change. For example, an EWS for arboviruses could inform decisions about when and where to deploy public health interventions to prevent an epidemic in the context of an impending anomalous climate conditions [25].

The key ingredient for embedding climate services in public health operations is creating an enabling environment for partnership with different stakeholders. This is done by identifying the common priorities, needs for research, and by building necessary capacities for understanding among climate and public health stakeholders and researchers [26,27].

Stakeholders from the health and climate sectors ideally play a central role in the development and implementation of a forecast model through an iterative engagement process with modelers and other scientists [28,29]. These stakeholders and other end-users of climate information are a diverse group of actors with distinct needs and interests [30,31]. Morss et al. [30] report insightful lessons learned as scientists who attempt to communicate flood risk to the public sector. They state, “Decision makers are not a coherent entity, but a collection of individuals, each of whom uses different information to address different goals in a unique context.” Climate services should be developed with a realistic understanding of the present-day public sector capacity, which may be constrained by resources, information, prior experiences, and other actors or institutions [32]. To guide this process, the Global Framework for Climate Services (GFCS) was designed as the policy mechanism to support the development of climate services for the health sector and other key sectors [33]. The GFCS aims for stakeholder engagement between health and climate actors at all levels to promote the effective use of climate information within health research, policy and practice [33].

Prior studies have focused on health sector perceptions of the effects of climate change and variability on overall health but have not assessed their perceptions of the potential role of climate services. Studies from the United States and Canada analyzed the perceptions and engagement of public health practitioners in the context of long-term climate change and impacts on health in general [34–38]. One study from China assessed health sector perceptions of dengue and climate change and identified a gap between climate change perceptions and behaviors/actions to reduce climate change risks [39]. In the Caribbean, a study found that health providers perceived mosquito-borne disease as increasing due to changing seasonal patterns [40], whereas another study found that health practitioners had limited understanding of the effects of climate variability on health [41]. Climate practitioners in Jamaica were generally aware of the health implications of climate change for heat stress, respiratory diseases, and vector borne diseases [42]. However, few studies (primarily for malaria early warning systems, MEWS, e.g. [43–45]) address both climate and health sector needs and interests with respect to climate-driven epidemic forecasts.

To address this gap, we conducted a study in the eastern Caribbean where we focused on four key areas, based on the GFCS health exemplar goals [33]:

1. What are the perceptions of climate-health or climate-arbovirus linkages?
2. Who are the key actors engaged in climate-arbovirus surveillance and control, and how can communication and partnerships amongst these actors be strengthened?
3. What are the current capabilities of the health and climate sectors to implement a climate-driven arbovirus EWS, and what capacities need to be strengthened so that the health sector can effectively access, understand and use climate/weather information for decision-making?
4. What climate/weather data are currently used by the health sector for arbovirus control, what added value does it provide, and how can climate/weather data be effectively tailored for arbovirus control operations?

## Methods

### Ethical statement

The study protocols were reviewed and approved (or deemed exempt) by the Institutional Review Board (IRB) of the State University of New York Upstate Medical University, the IRB of the University of the West Indies, Cave Hill Campus, on behalf of the Ministry of Health of Barbados, and the Ministry of Health and Environment of Dominica. No informed consent was required, as all participants were adults (≥18 years of age), were public sector employees, and no identifying information was gathered.

### Study sites

**Fig 1:**
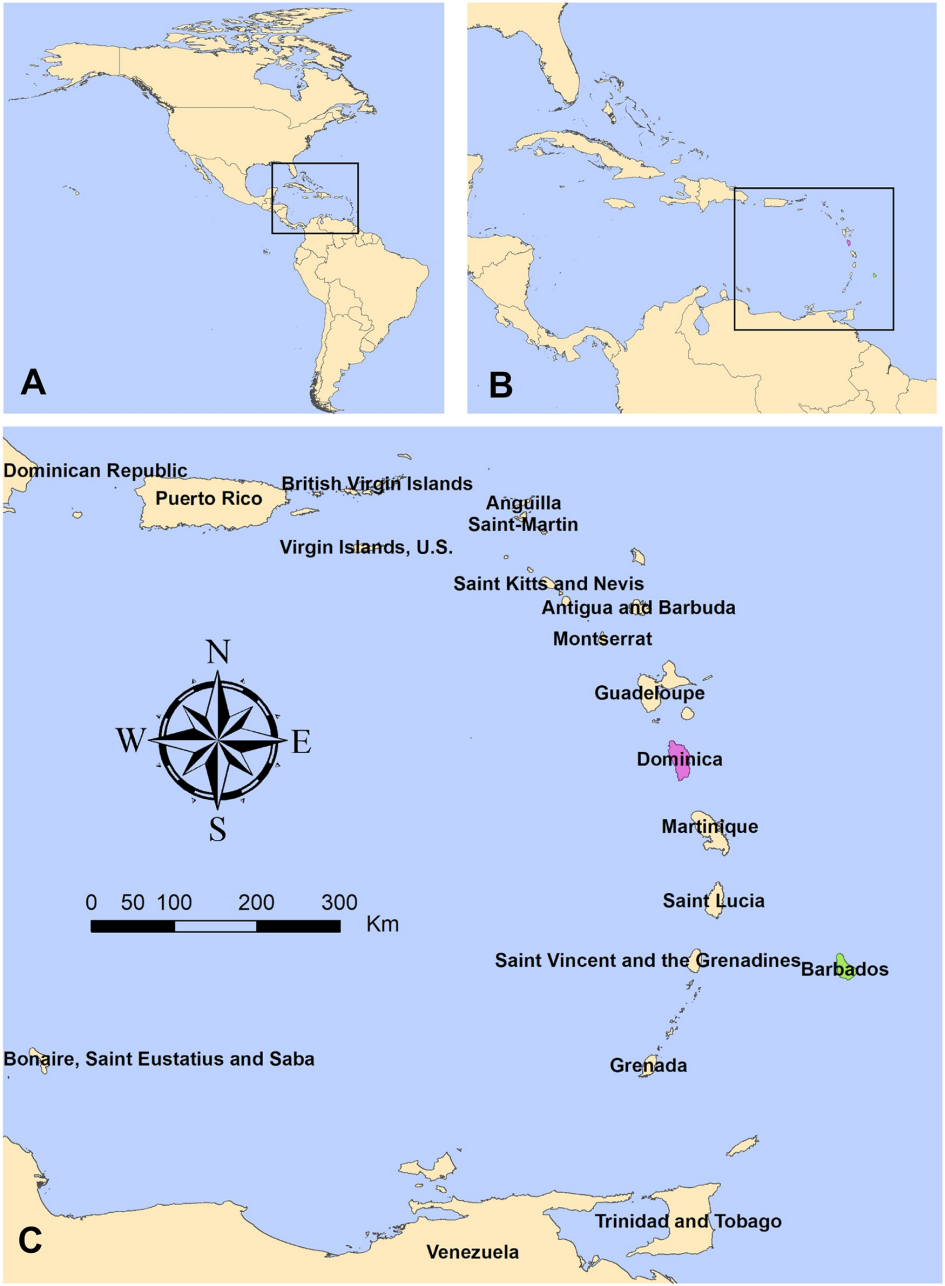
Map of the study region. (A) Location of the Caribbean region within the Americas, (B) Archipelago of islands making up the Caribbean and their location within Meso-America. (C) Location of Dominica (purple) and Barbados (green) within the islands in the region. This map was created using freely available country boundary datafrom GADM.org, rendered in ArcGIS. Image files were created using GIMP freeware.

This study focused on the perspectives of health and climate stakeholders from the countries of Barbados and Dominica (Fig 1), SIDS in the eastern Caribbean, as well as regional Caribbean stakeholders. There is a high burden of arboviral diseases in both countries [46–50] (Table 1). Barbados and Dominica were selected because of the regional and national interest in building on previous projects, wherein the health sector identified climate services as a top priority for the management of arboviruses [51,52].

**Table 1:** Arboviral disease cases in Barbados and Dominica.

	Barbados	Dominica
Mean annual dengue cases (2012–2016) [53]*	2,274	169
Mean annual dengue incidence (2012–2016) per 10,000 people [53]*	80.0	23.5
Total chikungunya cases since 2013 [54]	1,833 suspected 114 confirmed	3,590 suspected 173 confirmed
Total Zika cases since 2016 [48,49]	705 suspected 150 confirmed	1,263 suspected 79 confirmed

*Includes suspected and confirmed cases

Barbados (pop. 284,996; land area: 439 km^2^) has a service-based economy, with tourism accounting for 12% of the gross domestic product (GDP). Barbados is a water scarce country. Droughts threaten to reduce the already limited freshwater resources [55], and tourism is a water-intensive sector. The Ministry of Health is responsible for arbovirus and vector surveillance and control.

Dominica (pop. 73,543; land area: 750 km^2^) is characterized by abundant freshwater resources, forest and rugged terrain. Eco-tourism is becoming increasingly significant to its economy. The country was devastated by Hurricane Maria in September 2017, a category 5 hurricane that damaged 90% of buildings, resulting in USD 1.3 billion in damages, the equivalent of 224% of Dominica’s GDP in 2016 [56]. This study was conducted in the months prior to Hurricane Maria. The Ministry of Health and Environment is responsible for arbovirus and vector surveillance and control.

The national health agencies of both countries are supported by the Caribbean Public Health Agency (CARPHA) and the Pan American Health Organization (PAHO), the regional arm of the WHO. Each country has its own national meteorological and hydrological service (NMHS) supported by the Caribbean Institute for Meteorology and Hydrology (CIMH), the technical arm of the Caribbean Meteorological Organization (CMO). Details regarding the mandates and capabilities of the public health and climate national and regional organizations, with respect to arbovirus and vector surveillance and control, and climate monitoring and forecasting, are provided in S1 Text.

### Surveys and interviews

We collected data from key stakeholders from the climate and health sectors spanning senior leadership, managers, and expert practitioners. Stakeholders from the health sector were engaged in arbovirus epidemiology, vector control, or environmental health, at national and regional (Caribbean) agencies. Stakeholders from the climate sector were individuals involved in the development of climate services for the Caribbean region and managers/practitioners from NMHSs. Local leaders of the climate and health sectors assisted in identifying initial interviewees. Subsequent interviewees were identified through snowball methodology, whereby interviewees were asked to identify 2–3 additional stakeholders. We determined that we had effectively sampled all key stakeholders when no new names were identified; this was feasible given the relatively small size of the climate and health sectors in Dominica and Barbados.

A survey instrument was developed for health sector stakeholders. Questions were informed by prior large-scale surveys of health practitioner perceptions of climate change impacts on health conducted in the United States, called “Are We Ready?” [35–37], as well as studies by Paterson et al. [38] and Gould and Rudolf [34]. We collected basic demographic information and tailored the questions to focus on health sector perceptions of climate variability and arbovirus risk factors, perceptions of the public health sector response to climate variability, current use of climate information, how they prefer to interact with and receive EWS information, the current strengths and weaknesses of their department with respect to the implementation of an arbovirus EWS, and their training needs. In the survey, we defined climate variability as, “short-term changes in climate that occurs over months, to seasons, to years. This variability is the result of natural, large-scale features of the climate, often related to El Niño or La Niña events. Examples include floods, multi-year or seasonal droughts, heat waves, hurricanes or tropical storms.”

Printed surveys were distributed to health sector stakeholders at national vector control, environmental health, and epidemiology offices, as well as those who participated in national workshops on the development of climate services for arboviruses in Barbados and Dominica in April 2017. The workshop in Dominica was organized by the CIMH and the Ministry of Health and Environment (6 health sector participants). The workshop in Barbados was organized by the PAHO and the CIMH (21 health sector participants). Survey responses were entered into an online digital database using Qualtrics and responses were tabulated.

An interview instrument was developed for stakeholders from the climate and health sectors. Questions in the interview and survey were similar so that we could triangulate and validate the responses. We also asked which organizations they had partnered with to manage vector borne diseases, which organizations they would like to partner with, how climate and health fit within their current institutional priorities/mandates/competencies, and what strategies would stimulate collaboration between the climate and health sectors. In interviews with program directors, we asked additional questions about available climate and arbovirus/vector data and information, as well as arbovirus and vector surveillance and control strategies (see institutional competencies in S1 Text).

Project investigators interviewed stakeholders from the climate and health sectors through in-person meetings or via Skype in April and May 2017. Interviews were audio recorded following permission from interviewees. Recordings were transcribed and coded by project investigators to identify responses by research topic, country/region and sector [57,58].

During the Barbados national workshop, we conducted an exercise where health (n = 21) and climate sector (n = 6) participants were divided into small groups that included representatives from both sectors. Groups were asked to respond to different forecast scenarios (2-week, 3 month, and 1-year forecasts of *Ae. aegypti* larval indices and dengue incidence). They were asked to identify the actions that they would take in response to alerts at each time scale, and they discussed the utility of the different forecasts. As with interviews, responses were audio recorded, transcribed, and coded.

The study instruments were reviewed and tested by local collaborators, as well as the research team, prior to implementation. Instruments are available in S2 and S3 Texts.

## Results

We surveyed 32 individuals from the health sector and interviewed 41 individuals from the climate (n = 10) and health (n = 31) sectors. Respondent demographics are shown in Table 2. Several individuals participated in both interviews and surveys; however, the exact number is unknown since identifiable information was not collected from surveys.

**Table 2:** Demographics of survey and interview participants.

Responses	Survey % (n)	Interview % (n)
Total respondents	32	41
Female	72% (23)	56% (23)
Male	28% (9)	44% (18)
*Jurisdiction*		
Barbados	63% (20)	37% (15)
Dominica	31% (10)	41% (17)
Regional	6% (2)	15% (6)
*Sector*		
Health sector	100% (32)	76% (31)
Climate sector	**	24% (10)
*Range Age*		
18–30	9% (3)	*
31–40	25% (8)	*
41–50	31% (10)	*
51–65	25% (8)	*
> 65	3% (1)	*
No response	6% (2)	*
*Level of Education*		
Associate’s degree	16% (5)	0% (0)
Bachelor’s degree	22% (7)	2% (1)
Master’s degree	59% (19)	20% (8)
MD or PhD degree	0% (0)	15% (6)
No response	3% (1)	61% (25)
*Time working in sector*		
1–5 years	3% (1)	0% (0)
6–11 years	13.5% (4)	2% (1)
12–15 years	16% (5)	5% (2)
> 15 years	62.5% (20)	20% (8)
No response	6% (2)	76% (31)

*Data not gathered

**Only individuals from the health sector were surveyed

### (1) What are the perceptions of climate-health or climate-arbovirus linkages?

#### Perceptions of climate variability and health impacts

In surveys, health practitioners were asked to respond to a series of statements about the effects of climate variability on health in their jurisdiction and their ability to respond to these effects (Table 3). Most agreed that their jurisdiction is experiencing an increased risk of diseases transmitted by *Ae*. *aegypti* due to climate variability and that the risk will increase in the future. Survey respondents were worried about the effects of climate variability on health, and they agreed that this is an urgent problem in their jurisdiction. Although two thirds agreed that there are options or solutions to reduce the effects of climate variability on health, they disagreed that they had sufficient resources and expertise to assess the impacts of climate variability on health and to protect residents in their jurisdiction.

**Table 3:** Perceptions of climate variability impacts on health reported by survey respondents. Results shown as % (n). This most frequent response per question is highlighted in bold. Adapted from [35–37].

Questions	No response	Don’t know	Disagree*	Neither agree nor disagree	Agree*
My jurisdiction is currently experiencing one or more serious public health problems as a result of climate variability.	6% (2)	3% (1)	13% (4)	9% (3)	**69% (22)**
My jurisdiction is currently experiencing an increased risk of diseases transmitted by *Aedes aegypti* due to climate variability.	3% (1)	6% (2)	3% (1)	9% (3)	**78% (25)**
In the next 20 years, my jurisdiction will experience increasing risk of diseases transmitted by *Aedes aegypti* due to climate variability.	3% (1)	13% (4)	3% (1)	0 (0)	**81% (26)**
I am worried about the impact of climate variability on the health and well-being of people in my jurisdiction.	3% (1)	0% (0)	0% (0)	3% (1)	**94% (30)**
The effects of climate variability on the health of people in my jurisdiction is an urgent problem.	3% (1)	3% (1)	0% (0)	13% (4)	**81% (26)**
There are options/solutions to reduce the effects of climate variability and to improve the health of people in my jurisdiction.	3% (1)	3% (1)	16% (5)	13% (4)	**66% (21)**
The people in my jurisdiction are worried about the effects of climate variability on their health and wellbeing.	6% (2)	3% (1)	22% (7)	13% (4)	**56% (18)**
My health department currently has ample expertise to assess the potential public health impacts associated with climate variability that could occur in my jurisdiction.	3% (1)	0% (0)	**41% (13)**	19% (6)	38% (12)
Dealing with the public health effects of climate variability is an important priority for my health department.	6% (2)	0% (0)	13% (4)	22% (7)	**59% (19)**
I am knowledgeable about the potential public health impacts of climate variability.	3% (1)	0% (0)	16% (5)	3% (1)	**78% (25)**
The other relevant senior managers in my health department are knowledgeable about the potential public health impacts of climate variability.	13% (4)	3% (1)	19% (6)	13% (4)	**53% (17)**
My health department currently has ample expertise to create an effective plan to protect local residents from the health impacts of climate variability.	6% (2)	6% (2)	**34% (11)**	22% (7)	31% (10)
My health department currently has sufficient resources to effectively protect local residents from the health impacts of climate variability.	9% (3)	6% (2)	**57% (18)**	19% (6)	9% (3)
My health department is able to effectively communicate the health impacts of climate variability to local communities.	9% (3)	0% (0)	31% (10)	19% (6)	**41% (13)**

*Agree and strongly agree were combined into one category, as were disagree and strongly disagree

#### Climate and non-climate risk factors for Aedes-transmitted diseases

Survey respondents were asked to identify the relative importance of climate and non-climate risk factors in triggering epidemics of diseases transmitted by *Ae*. *aegypti* (Table 4). Non-climate risk factors were identified as more important overall than climate risk factors. The key non-climate risk factors, in order of importance (% very important or important), were the introduction of new viruses (100%), water storage (97%), human movement (91%), limited community engagement (91%), insecticide resistance (88%), and insufficient resources (88%). Climate risk factors, in order of importance (% very important or important), were heavy rainfall (91%), El Niño or La Niña events (72%), drought conditions (56%) and warm air temperatures (51%).

**Table 4:** The relative importance of factors that trigger epidemics of diseases transmitted by *Aedes aegypti*, as reported by survey respondents. Results shown as % (n) of survey respondents, ranked by risk factors identified as “very important.” The most frequent responses are marked in bold.

Categories	No response	Slightly important	Moderately important	Important	Very Important
Introduction of a new virus to a susceptible population	0 (0)	0 (0)	0 (0)	9.4 (3)	**90.6 (29)**
Water storage behavior	3.1 (1)	0 (0)	0 (0)	15.6 (5)	**81.3 (26)**
Insecticide resistant mosquitoes	0 (0)	6.3 (2)	6.3 (2)	18.8 (6)	**68.8 (22)**
Heavy rainfall	0 (0)	3.1 (1)	6.3 (2)	**46.9 (15)**	43.8 (14)
Human movement	0 (0)	3.1 (1)	6.3 (2)	**46.9 (15)**	43.8 (14)
Insufficient staff/resources for vector control	0 (0)	0 (0)	12.5 (4)	**43.8 (14)**	**43.8 (14)**
Lack of community knowledge and awareness	0 (0)	3.1 (1)	15.6 (5)	37.5 (12)	**43.8 (14)**
Limited community engagement/mobilization	0 (0)	3.1 (1)	6.3 (2)	**56.3 (18)**	34.4 (11)
Drought conditions	3.1 (1)	31.3 (10)	9.4 (3)	25 (8)	**31.3 (10)**
High-risk housing conditions	9.4 (3)	12.5 (4)	21.9 (7)	25 (8)	**31.3 (10)**
Low risk perception by communities	3.1 (1)	3.1 (1)	12.5 (4)	**50 (16)**	31.3 (10)
Economic barriers to mosquito control by households (e.g., cost of screens or insecticide)	0 (0)	9.4 (3)	31.3 (10)	**34.4 (11)**	25 (8)
El Niño or La Niña events	3.1 (1)	6.3 (2)	18.8 (6)	**50 (16)**	21.9 (7)
Warmer air temperatures	6.3 (2)	25 (8)	18.8 (6)	**31.3 (10)**	18.8 (6)

Regional and national interviewees were also asked to discuss climate and non-climate risk factors for arbovirus epidemics. They indicated that frequent (re)-introduction of viruses and vectors was associated with human movement between the islands due to trade and tourism. In Dominica, interviewees identified human movement between rural and urban areas as a risk factor. With respect to climate, interviewees identified the onset of the hot, rainy/wet season as a risk factor for arbovirus transmission, although they perceived that the linkages between rainfall and dengue fever have become less clear due to water storage practices. Two interviewees highlighted this contradiction,

“If the rain falls very heavily, within two weeks [we] expect to have an increase in number of cases. It’s always associated with rainfall.”

(Health Sector, Barbados)

“With these droughts, there doesn’t seem to be, in the last few years, a real dengue season.”

(Health Stakeholder, Barbados)

In Barbados, interviewees indicated that household water storage was associated with drought conditions and the resulting water scarcity. Another risk factor was the national legislation requiring that all new buildings greater than 1,500 square feet have rainwater storage receptacles as a drought adaptation strategy; however, the receptacles had become potential *Ae*. *aegypti* larval habitat. Interviewees indicated that the improper management of public utilities and infrastructure (e.g., telephone junction boxes, manhole covers, public wells, drains) had resulted in cryptic mosquito larval habitats that were difficult to locate and treat with larvicide during the wet season.

In Dominica, interviewees commented that water storage had increased following Tropical Storm Erika in 2015. When the storm damaged the piped water systems, people began storing freshwater in 55-gallon drums around the home. This behavior continued despite repairs to water systems. One interviewee described the effects of Erika,

“After the Tropical Storm Erika, everything just got a little more vulnerable than it used to be… it was just one downpour of rain that caused all of the destruction.”

(Health Stakeholder, Dominica)

In Dominica, interviewees noted that *Ae*. *aegypti* had expanded its range into higher elevation areas, where the mosquito had not been present historically.

#### Other effects of climate on health

Interviewees identified other ways that climate affected health in their jurisdiction. This included increased risk of morbidity due to the interaction of heat stress and diabetes associated with hotter days and nights, leptospirosis (*Leptospira* sp.) associated with flooding, and communicable diseases associated with relocation and crowding of people in shelters following tropical storms. Malnutrition was associated with droughts that reduced crop yields and warming ocean temperatures that caused fish kills. Respiratory problems (e.g., asthma) were associated with dry weather, dust and air pollution. Factors unique to Barbados included hypertension due to sea level rise and salt-water intrusion in the groundwater supply, reduced hygiene and *Pseudomonas* infections due to water scarcity and storage, skin cancer due to UV exposure, and water-borne diseases (e.g., gastroenteritis, *Salmonella*) associated with flooding. Factors unique to Dominica included loss of lives due to landslides associated with tropical storms, gastroenteritis associated with dry weather, and mental health morbidity in the elderly and other vulnerable populations who were relocated after tropical storms. One informant described the complex cascading impacts of climate on health,

“[During droughts] people are not able to go to their farms; they don’t have food and their nutrition suffers. They don’t have income … they cannot get their medications… So, its just the rippling effect.”

(Health Stakeholder, Dominica)

However, due to the lack of local research, interviewees recognized that most of these linkages were anecdotal or hypothetical, as summarized by one interviewee,

“So we have *not been able* to make a direct link between those diseases and climate variability and change; however, we know that there has been an increase as a result of climate variability… The data to make that linkage… is not really always available.”

(Health Stakeholder, Dominica)

### (2) Who are the key actors engaged in climate-arbovirus surveillance and control, and how to strengthen communication and partnerships amongst these actors?

#### Partnerships

**Fig 2:**
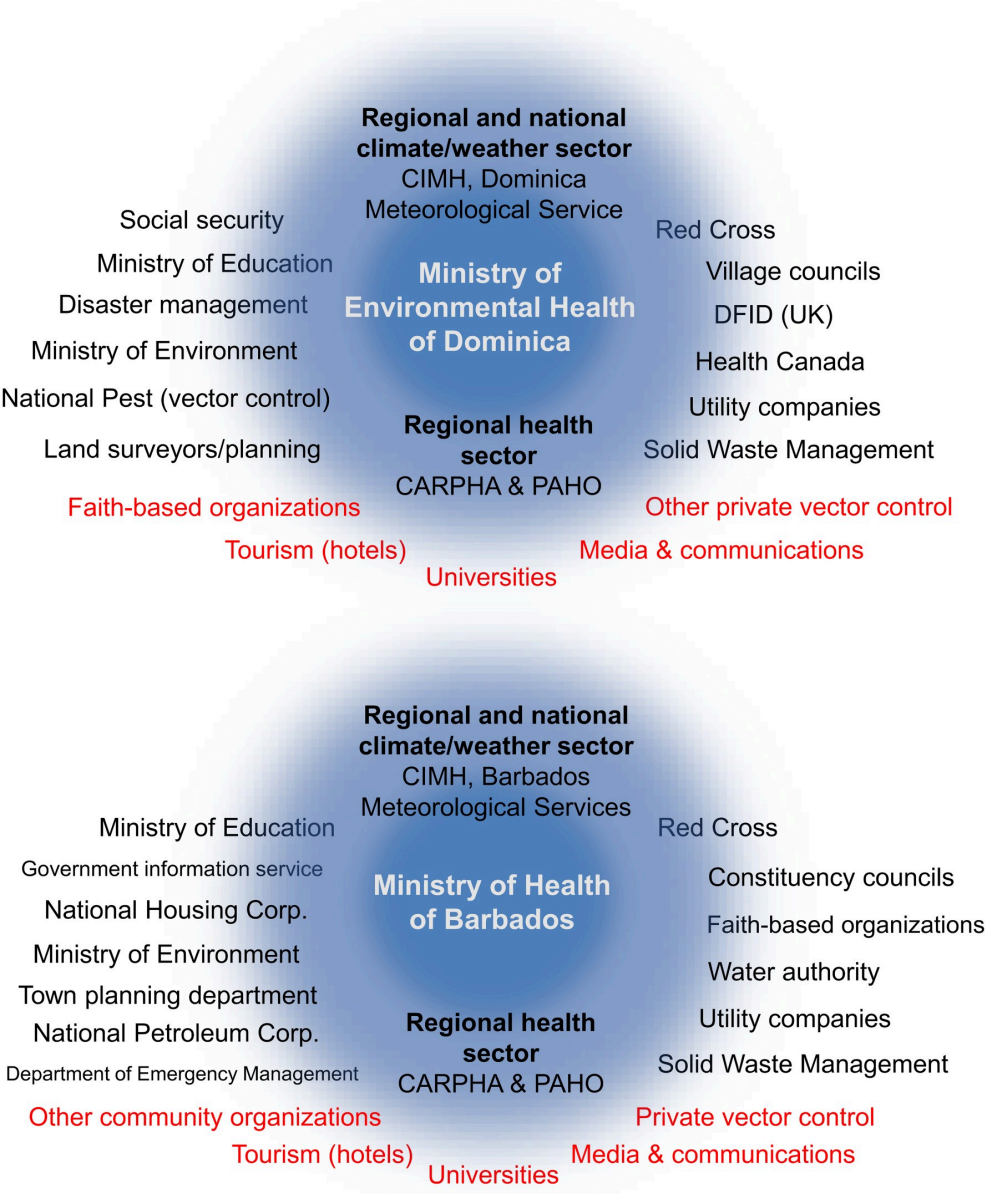
Stakeholder analysis. Organizations that partner with the health sector (in white) in Barbados and Dominica on issues related to vector control and climate services for health. Organizations in black are current functioning partnerships. Organizations in red are partnerships that need to be strengthened with the health sector.

Regional and national interviewees identified a network of agencies and funders engaged in climate-arbovirus surveillance and control (Fig 2). The key regional institutions were the PAHO, the CARPHA, and the CIMH. The Red Cross was the most frequently mentioned non-governmental organization (NGO). The health sectors engaged periodically with their respective NMHS on specific projects; however, there were no formal collaborations. Understanding and mitigating the effects of climate on health were relatively high priorities in the health sector, but climate and health was not yet a mandate for the national health or climate sectors (S1 Text). As a result, it was difficult to allocate resources (e.g., personnel, funding) to this area.

Interviewees indicated that the key partnerships to be strengthened were the private sector (tourism, vector control companies, media), academic institutions, and civil society organizations. Interviewees proposed that the tourism sector, hotels, could support vector control, given their concern with reducing disease risk for tourists. In Barbados, health interviewees stressed the need to regulate insecticide use by private vector control companies to reduce the risk of insecticide resistance in mosquito vectors. Private media were identified as partners who could assist with public health messaging, and civil society organizations could help with community mobilization during health campaigns. Interviewees identified the need for stronger collaborations with academic partners to generate evidence to inform public health decision-making.

#### Collaboration strategies

Regional and national interviewees identified six strategies to strengthen the communication and partnerships amongst these actors.

First, they highlighted the importance of an integrated approach to the development of climate services for health spanning research, operations, a platform for data and knowledge sharing, outreach, awareness raising, education, an in-country response, and mitigation plans and policies.

Second, interviewees emphasized the importance of engaging senior leaders from the health sector to raise the profile of climate and health on the health agenda, and to ensure that actions are driven from the top-down.

Third, they highlighted the importance of formal collaboration agreements amongst climate, health, and other sectors. Regional stakeholders mentioned the multi-lateral agreements recently signed amongst the CIMH, the CARPHA and other regional Caribbean agencies (see S1 Text for details). Interviewees indicated that collaboration agreements would allow them to co-develop and co-deliver climate services for the health sector. The agreements signaled a strong commitment from institution directors and an understanding of mutual benefit.

Fourth, they suggested that national committees on climate and health be established to specify the work that would be done jointly, the roles of each partner, a timeline for an operational plan, and standard operating procedures (SOPs) with a framework for communication, and reporting guidelines. Development of data sharing protocols between the climate and health sectors was identified as a priority given the sensitivity of sharing health information.

Fifth, interviewees indicated the importance of creating shared spaces for dialogue between the climate and health sectors, such as regional and national climate and health forums. An interviewee from the regional climate sector stated,

“Just sitting with people in the sectors makes such a big difference… Understand them, what drives them, what are their needs? Because we might think they need something they don’t… Sometimes it’s about forgetting yourself and putting yourself in the other person’s shoes to really figure out what the need is about. That’s true engagement.”

(Climate Stakeholder, Regional)

This engagement would facilitate functional working relationships and increase the trust among people in both sectors, allowing sectoral stakeholders to learn about the needs and perspectives of the other, what information can be shared, and the resources available to help each other. One regional interviewee stated,

“Once we build the trust, then we build the network, then we can see what the willingness to collect, to centralize, to digitize, and to share the data really is.”

(Climate Stakeholder, Regional)

For example, interviewees suggested that the MoH could partner with their NMHS so that new weather stations are placed in areas that are strategic for arbovirus surveillance, and the NMHS could participate in epidemiological surveillance meetings of the MoH.

The sixth strategy (proposed by regional climate interviewees) was that climate services for health be framed as a national development priority, increasing buy-in from decision makers and funding from international development agencies. One regional interviewee stated,

“I think people will embrace climate and health… [it is] a real sustainable development goal… Health has always been a critical sector.”

(Climate Stakeholder, Regional)

### (3) What are the current capabilities of the health and climate sectors to implement a climate-driven arbovirus EWS? What capacities need to be strengthened?

#### Current capabilities

**Fig 3:**
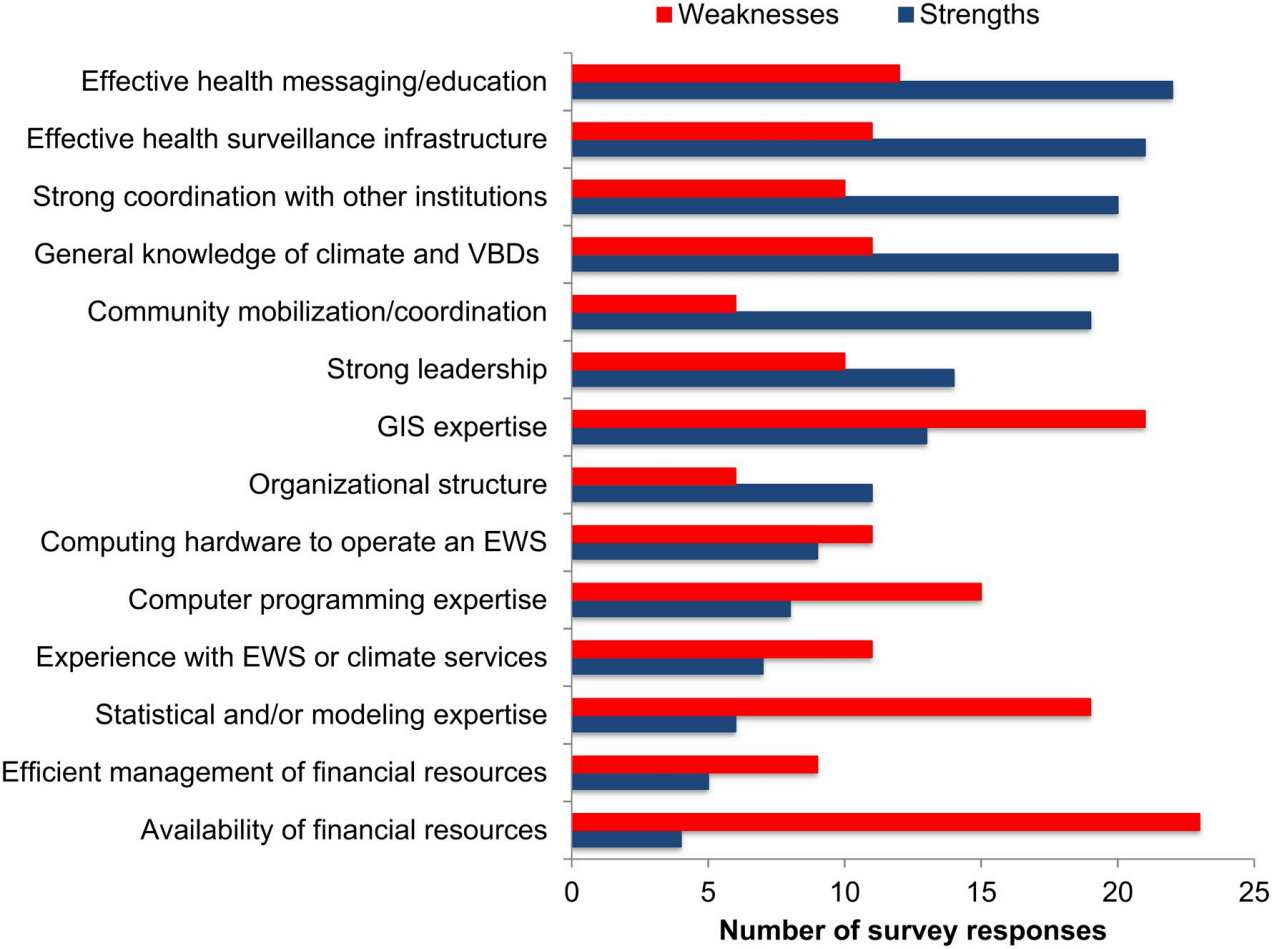
Perceptions of the strengths and weaknesses of the health sector with respect to the capacity to implement an early warning system for arboviral diseases. EWS = early warning system, GIS = geographic information system, VBDs = vector borne diseases. Results shown as the number of health sector survey respondents (n = 32).

Health sector survey respondents were asked to identify the strengths and weaknesses of their institution with respect to the implementation of an arbovirus EWS (Fig 3). The top strengths were effective public health messaging to communities, effective health surveillance infrastructure, knowledge of the effects of climate on vector borne diseases, strong coordination with other institutions, and community mobilization. The top weaknesses, or areas to be strengthened, were the availability of financial resources and expertise in geographic information systems (GIS), statistics, modeling, and computer programming (see S1 Table for software expertise in health departments and S2 Table for preferred training activities). The need for training in data analytics was summarized by a regional health sector interviewee,

“Having got the data, how do we use it? What do we use it for?”

(Health Stakeholder, regional)

NMHS interviewees confirmed that they had limited resources to implement climate services for health. They emphasized the need to learn more about the health sector end-user needs. In Dominica, the NMHS identified the need for basic resources to increase their monitoring and forecasting capacities, including a staff meteorologist, adequate transportation to and from meteorological stations, financial resources, instrumentation, and improved security to prevent vandalism to the meteorological stations. They stated,

“We use our personal vehicles, but some of the areas are a bit challenging, and we are two females, so sometimes …depending on where we are going, we need somebody else to go with us, for security”

(Climate Stakeholder, Dominica).

#### Training needs

Regional interviewees highlighted the importance of training, nurturing, and retaining a cohort of practitioners with expertise in climate and health to ensure the sustainability of an arbovirus EWS. National health sector interviewees emphasized the need to increase their skills in modeling and data analysis through technical workshops on how to use climate information, data, models and other tools to predict epidemics. They identified the need for training on climate and health linkages, greater understanding of climate services for health, how to use climate services for health during emergencies/disasters, and how to communicate the effects of climate on health to local communities. National health sector interviewees suggested that training activities be practical and interactive, such as workshops where multisectoral teams respond to simulations of epidemic warnings.

Regional and national health sector interviewees expressed an urgent need for training on geographic information systems (GIS) to visualize and analyze arbovirus, vector, and climate information. They found that GIS was a highly effective tool that allowed them to use field data to make informed decisions, and to communicate risk information back to the public.

“It is so much easier, better, to use maps when you are doing presentations. Especially if you are doing something with the public where you can actually show them their community and say, ‘There you have breeding sites. There is where you have the problem.’ And they can actually see it. You can actually show it to them.”

(Health Stakeholder, Dominica)

Interviewees from both countries highlighted the need for better data collection and storage practices in the health sector in order to create high-quality, long-term datasets.

#### The need for local research

Regional and national interviewees stressed the need to increase local research capabilities, in part by strengthening collaboration with universities (Fig 2). Interviewees from the Barbados NMHS indicated that they had limited experience with climate research, and that this was an area that they were interested in expanding. National health sector interviewees displayed a high level of field experience and local knowledge but indicated that they had little knowledge of local empirical studies that could inform their decision-making and planning processes. As stated by one regional health sector interviewee,

“We want more evidence-based decision-making. We want data… That’s priority #1… to get the evidence.”

(Health Stakeholder, Regional)

Regional interviewees recommended conducting case studies or demonstration projects to generate local evidence on climate-health linkages. They suggested focusing these investigations and interventions at the medium-term (climate variability) time scale (e.g., seasonal variation, year-to-year variation in extreme climate events), rather than the long-term (climate change) time scale. Interviewees highlighted that a collaborative research process with investigators from the climate and health sectors would facilitate data sharing, build trust, and foment a culture of research on climate and health.

### (4) What climate/weather data are currently used by the health sector for arbovirus control, what is the added value, and how can climate/weather data be effectively mainstreamed for arbovirus control operations?

#### Use of climate information

Health sector survey respondents were asked about their current use of climate information (S3 Table). Two thirds of respondents indicated they had received general information on the effects of climate on vector borne diseases, and half of the respondents confirmed that climate information was used for some level of planning for disease and vector control interventions. They were unsure as to whether an EWS for arboviruses existed in their jurisdiction, but most indicated that climate information was not part of existing epidemiological warning systems.

National and regional interviewees confirmed that the arbovirus alert systems in Dominica and Barbados were based solely on epidemiological surveillance. The health sector and PAHO issue an alert when the number of reported cases surpasses a pre-determined threshold established by the historical average for the same week or month (see Lowe et al. [11] for details). They indicated that the current system did not provide sufficient lead-time to effectively reduce the threat of an epidemic.

National interviewees described the current use of climate information for arbovirus control. The health sector considers wet/dry seasons and extreme climate events when planning vector control programs, for example, by increasing larviciding efforts at the onset of the wet season or increasing community campaigns on safe water-storage during droughts. Occasionally, the health sector requests climate data from their NMHS (typically shared as Excel files). However, the health sector does not formally incorporate climate information, such as seasonal climate forecasts, into their planning process. Overall, climate information was reported to play a minor role in decision-making, which was instead driven by policies, regulations, and specific competencies of the organizations.

#### Forecast scenarios

At the national workshop in Barbados, health and climate stakeholders were asked to identify the interventions they would implement if they were provided with short (two week), medium (three month) and long-term (one year) forecasts of vector abundance and dengue incidence (S3 Text). They unanimously stated that disease incidence forecasts would be more effective than vector forecasts in garnering the political attention necessary to mobilize resources to implement preventative interventions. With a short-term forecast, the health sector would increase education, community mobilization, and larval source reduction, especially in known hotspots. With a medium-term forecast, the health sector would be better able to plan with stakeholders, mobilize the field team, look at trends, and create bulletins for community mobilization. With a long-term forecast the health sector could better lobby with health sector leadership and the Minister of Finance for the needed financial support, allowing for more effective budgeting. They would be able to monitor and evaluate interventions and conduct a needs assessment to inform planning. They would also be able to procure diagnostic reagents and supplies for the national reference laboratory, a process that can take up to six months in Barbados. Although the workshop participants identified meaningful interventions at each time scale, they preferred the three-month forecast as indicated in the following,

“A year can feel like a long time away. With three months, there will be a sense of urgency and you can do meaningful activities, although there might not be new resources.”

(Health Stakeholder, Barbados)

#### Added value of climate services

Both regional and national health sector interviewees highlighted the ways in which climate services would improve their planning for arbovirus interventions. By integrating climate and/or disease forecasts into their seasonal and annual planning processes, they felt that they could be proactive and effective at preventing outbreaks, as described by this interviewee,

“We know we have *Aedes*, we know we have the threat, but its only when outbreaks happen, we start scrambling around to do things. So I think if we can put mechanisms in place, long in advance, then we can have more success in dealing with outbreaks. Or we can even prevent outbreaks.”

(Health Stakeholder, Dominica)

Interviewees indicated that climate services have to provide reliable information early enough such that the health sector can target control efforts in high-risk areas during certain times of the year. This would result in a more efficient use of limited financial and human resources, as described by this interviewee,

“When you know that there is an impending threat, you would come up with specific activities that you would conduct. It doesn’t necessarily mean that those activities would be at a higher cost, but you can be more specific… It will be easier for us to respond to an impending threat, instead of running around.”

(Health Stakeholder, Dominica)

Interviewees indicated that forecasts of disease risk could be used to inform hospitals about staffing needs, stocking of medicines and laboratory diagnostic reagents, and the development of targeted educational materials for the public. They suggested that warnings be communicated to the public through social media and other outlets to motivate community mobilization for preventative practices. Health sector interviewees indicated that they would feel more motivated and inspired in their day-to-day work if they could see how the epidemiological and vector data that they collected was used to inform decision-making.

#### Mainstreaming climate services

Health sector survey respondents were asked how they would prefer to receive information from an arbovirus EWS. The top responses were a climate and health bulletin (91%), an interactive GIS platform (66%) and internal meetings within their departments (59%) (S4 Table). Interviewees were also asked to identify climate services that would improve their day-to-day work related to arboviruses. National health sector interviewees confirmed that they were interested in utilizing the Caribbean Health Climatic Bulletin launched in 2017 by the CIMH, the CARPHA and the PAHO. The bulletin qualitatively summarizes potential health impacts for a three-month period based on seasonal climate forecasts (https://rcc.cimh.edu.bb/health-bulletin-archive/). Health sector interviewees also reiterated that a GIS platform would allow them to integrate, analyze, and visualize real-time information on vectors and disease risk in relation to rainfall, temperature, and other meteorological variables. Wind speed and wind direction forecasts were also identified as useful information to inform insecticide fogging operations. Regional stakeholders highlighted the importance of creating a multi-country health and climate data repository that would allow for a regional epidemic EWS. National interviewees summarized the health sector needs in the following,

“We need it [climate services] packaged in such a way that the health professional would understand. Pick it up, and look at it, and understand it.”

(Health Stakeholder, Dominica)

“Decision makers at the policy level are not healthcare providers. They are administrators, they are politicians, and we need to help them. We need to feed them [decision makers] with the kind of information they can understand, and [so] they can feel comfortable making decisions.”

(Climate and health workshop participant, Barbados)

## Discussion

A climate-informed epidemic EWS is a potentially powerful tool to guide public health decision making. Our study confirms that an arbovirus EWS would benefit the Caribbean health sector–a region that is highly vulnerable to extreme hydrometeorolgical events and arboviral disease outbreaks. However, we found that the development of climate services for arboviral diseases will require stronger partnerships, strengthening of local capacities, and greater investment in local research on climate-health linkages. Our findings are echoed by Huang et al [59], who emphasize that health sector adaptation to climate change requires both the implementation of adaptation actions (e.g., climate-driven EWS for arboviruses) and adaptive-capacity building.

In this study we identified strategies and opportunities to initiate a successful process of joint collaboration between the climate and health sectors (see S4 Text). This partnership is critical to ensure commitment and ownership by different stakeholders and end-users as climate services are developed. We found that the sustainability of these initiatives will require the political will to establish climate services for health as a mandate in the NMHS and health sectors, allowing them to work in interdisciplinary teams. Beyond the climate and health sectors, we identified a complex web of institutional actors who can engage strategically in the development of climate services for health. This finding highlights the importance of intersectoral collaborations, a key element of Integrated Vector Management for arbovirus control [60].

We found that health sector stakeholders demonstrated concern, awareness and a high-level understanding of the impacts of climate variability on arboviruses and health in general. People from the health sector identified an increased risk of arboviral diseases associated with climate variability. In this region, climate variability is associated with droughts and tropical storms; interviewees identified both as having the potential to increase the risk of arboviral disease outbreaks, as confirmed in prior research [11]. With an increasing number of potential arboviral disease threats [61] (dengue, chikungunya, Zika, Mayaro) and emerging insecticide resistance [62] in *Ae, aegypti*, it is possible that health stakeholders perceived that extreme hydrometeorological events and warmer temperatures may have a greater epidemiological impact today and in the future than in the past, when dengue was the only known arbovirus in circulation. It is also possible that survey respondents intermixed the concepts of climate variability and climate change.

Prior studies suggest that climate-health awareness has increased in the Caribbean over time. Earlier studies found that there was limited knowledge about climate and health linkages amongst nurses and doctors in private and public sectors [41]. More recent studies in the Caribbean confirm a relatively high level of awareness and concern amongst health-care providers [40], similar to studies in the U.S. [35,37]. Several capacity building initiatives undertaken by regional health institutions have likely contributed to higher levels of climate-health awareness over time.

Our results also highlight the importance of considering non-climatic drivers of arbovirus epidemics (e.g., human movement, insecticide resistance, and community mobilization), which were perceived to be as important or more important than climate factors in determining arbovirus outbreaks. Prior studies have highlighted the importance of human movement in propagating arbovirus transmission [63,64], including in island settings [65]. Insecticide resistance in *Ae. aegypti* is a major challenges for the health sector, given their reliance on chemical control to reduce disease transmission [66] and lack of regulation of private vector control companies in Barbados. Community mobilization for arbovirus control is an ongoing challenge throughout dengue-endemic regions [67], requiring a nuanced understanding of local community perceptions and behavior [68,69]. Following several years of arbovirus epidemic alerts, Caribbean communities may experience message fatigue [70] and be less responsive to future epidemic alerts. While community perceptions and communication were beyond the scope of this study, these elements should be considered in the development of an EWS.

Despite high levels of awareness, our findings suggest that the climate and health sectors do not feel ready to develop and implement an EWS or other adaptation measures due to limited institutional capacity (resources and expertise). This was also found in prior studies of health care professionals in the U.S. and Canada [34–38,59]. Health stakeholders stressed the need to increase analytical capabilities, such as GIS skills. User-friendly analytic tools/instruments that combine health and climate information could be developed for the health sector for use in routine reporting activities. We found that the national climate sectors (NMHSs) also faced severe capacity limitations. Prior studies have noted the embryonic status of the application of climate science in the Caribbean health sector [24], with only one NMHS offering specialized climate information services for the health sector [71]. The capacity of NMHSs is especially limited in SIDS like Barbados and Dominica [71]. In recent research, representatives from the NMHSs recommended transitioning from a designation of NMHS to become “National Climate Services Centers (NCSC),” which would facilitate the development of climate services at the national level [71].

Our results confirm that climate information is neither routinely applied nor used in planning arbovirus/vector interventions in Barbados or Dominica, notwithstanding major advances in climate science and climate-health research globally. The operational co-production of tools and products, such as the quarterly Caribbean Health Climatic Bulletin is a noteworthy first step. The bulletin includes qualitative expert statements on probable health risks associated with seasonal climate forecasts (three months ahead). However, there is significant scope for the development of the next generation of climate services that focus on quantitative probabilistic forecasts of disease risk [24]. One of the most promising examples of a climate-driven dengue forecast model framework was recently described by Lowe et al. for Barbados [11]. The study found that dengue transmission in Barbados increased one month after a particularly wet month and five months after a drought event was observed, and the model was able to accurately predict outbreak versus non-outbreak months. Seasonal climate forecasts routinely produced by the CIMH could be incorporated in the model framework as an early warning tool. This could help the health sector to plan interventions that mitigate the impact of mosquito-borne disease epidemics in the region up to three months in advance—the ideal window of prediction identified by study participants.

### Limitations

When comparing the results of this study to prior studies on health sector perceptions of climate, one key difference is that our study focused on people working with arboviruses, environmental health, and climate, whereas other studies focused on health-care providers or public health professionals in general. However, given the relatively small size of the health sector in Barbados and Dominica, we interacted with most senior leadership in interviews and national consultations, in particular those involved with overall management of the public health sector, epidemiological programs, environment, climate change and health. Although we did consider a regional perspective, the results of this study may not be generalizable to all of the Caribbean. Country-level studies should be conducted to capture the nuances of local governance structures, disease epidemiology, and climate.

Our results were skewed towards the health sector perspective rather than the climate sector, given that more health sector stakeholders were interviewed, and only health sector stakeholders were surveyed. In part, this reflected that there were many more people working in the national health sectors than in the national climate sectors. On the climate side, our results were skewed towards the regional perspective, given that regional stakeholders had more experience with climate services for health.

### Conclusions

The results of this study provide recommendations to enhance an interdisciplinary dialogue and partnership within an active community of practitioners, decision makers, and scientists [33,72]. This study contributes to a broader effort to work collaboratively with regional and national health and climate stakeholders in the Caribbean to develop decision support models to predict arbovirus risk and to design effective warning and intervention strategies [24]. Overall, the appropriate involvement of stakeholders is a key element to identify users’ needs, to develop users’ capacities and to exploit existing capabilities. More information regarding national and regional policy opportunities are presented in S4 Text.

One of the key conclusions of this study is the need to strengthen the provider-user interface, as currently there is only limited consideration of the products needed by health sector users. Climate services for health can only become operational with the will and support of the climate and health sector institutions. At the same time, it is necessary to create appropriate ‘communities of practice’ and to emphasize the co-design of climate services products [73]. Final recommendations include:

1. To engage senior leadership in the establishment of collaboration agreements (MOUs) between the climate and health sectors, with a focus on climate services for health as a national development priority.
2. To strengthen the capacity of NMHS through their designation as National Climate Services Centers (NCSC) [71], allowing them to build capacity around the basic and operative aspects of climate services and to collaborate with health sector partners to promote climate services for health.
3. To strengthen partnerships with key sectors such as tourism (hotels), private vector control, universities, civil society groups, and private media.
4. National Adaptation Plans for Climate Change, including recent regional efforts to create Health National Adaptation Plans, may be an opportunity to include a policy or mandate for the inclusion of climate in health sector decision-making, and may be an opportunity to strengthen climate services, applying long-term scenarios for planning in health and other sectors (see S4 Text).
5. To strengthen health sector engagement in the region through annual forums focused on climate services and capacity building tailored to the health sector. This could build on existing regional climate meetings like the bi-annual Caribbean Climate Outlook Forum convened by the CIMH (see S4 Text).
6. To strengthen analytic capabilities in the health sector, and to develop data visualization tools for non-experts.
7. To support local research on climate-health linkages through stronger partnerships with academic institutions, particularly at the climate variability time scale.

## Supporting information

S1 Text Climate and health sector mandates and competencies. This document describes the mandates and competencies of regional (Caribbean) and national (Barbados and Dominica) climate and health sectors with respect to arbovirus and vector surveillance and control, and climate monitoring and forecasting. Information was gathered through face-to-face interviews with key stakeholders.
(DOCX)

S2 Text Interview and survey instruments. This document contains (1) an interview instrument used with climate and health decision makers, managers and expert practitioners, (2) supplemental interview questions regarding climate and health data, institutional mandates and competencies, and (3) a survey for health sector decision makers, managers, and expert practitioners.
(DOCX)

S3 Text Forecast scenarios discussed in the Barbados stakeholder workshop. This activity was conducted at a national consultation at the PAHO in Bridgetown, Barbados, in April 2017, with 27 representatives from the national Ministry of Health and Wellness (MoH) of Barbados, the Barbados Meteorological Services, the CIMH, and the PAHO. Participants were divided into small groups that included representatives from climate and health sectors. Groups were asked to respond to different forecast scenarios (2-week, 3 month, and 1-year forecasts of *Aedes aegypti* larval indices and dengue incidence). They were asked to identify the actions that they would take in response to alerts at each time scale, and they discussed the utility of a vector versus disease forecast. Results were identified by coding the transcripts of audio recordings.
(DOCX)

S4 Text National and regional policy opportunities. (DOCX)

S1 Table Types of software for which there is existing expertise in health departments as identified by health sector survey respondents. Results from surveys are shown as % (n).
(DOCX)

S2 Table Preferred training activities identified by health sector survey respondents. Results from surveys are shown as % (n).
(DOCX)

S3 Table Current use of climate information and early warning systems reported by health sector survey respondents. Results shown as % (n).
(DOCX)

S4 Table Preferred way of receiving information from an early warning system that predicts arbovirus epidemics as reported by health sector survey respondents. Results shown as % (n).
(DOCX)

## References

[1] Ebi KL, Lewis ND, Corvalan C. Climate variability and change and their potential health effects in small island states: information for adaptation planning in the health sector. Environ Health Perspect. 2006;114: 1957 10.1289/ehp.8429 17185291 PMC1764155

[2] PAHO/WHO. Climate change and health in small island developing states: A WHO special initiative in collaboration with UNFCC and the Fijan Presidency of COP-23. SIDS in the Caribbean Region [Internet]. Pan American Health Organization; 2018. https://www.paho.org/hq/index.php?option=com_docman&view=download&category_slug=technical-reports-9862&alias=46262-climate-change-and-health-in-small-island-developing-states-1&Itemid=270&lang=en

[3] Briguglio L. Small island developing states and their economic vulnerabilities. World Development. 1995;23: 1615–1632. 10.1016/0305-750X(95)00065-K

[4] Kelman I, West JJ. Climate Change and Small Island Developing States: A Critical Review. Ecological Environ Anthrolology. 2009;5(1).

[5] Caribbean Public Health Agency. State of Public Health in the Caribbean Region 2014–2016; Building resilience to immediate and increasing threats: vector-borne diseases and childhood obesity [Internet]. CARPHA; 2017. http://carpha.org/Portals/0/articles/documents/State-of-Public-Health-in-the-Caribbean-2014-2016.pdf

[6] Caribbean Public Health Agency. State of Public Health in the Caribbean Region: Inaugural Report [Internet]. CARPHA; 2013. http://carpha.org/downloads/CARPHA-State_of_Public_Health_Inaugural_Report_2013.pdf

[7] Caribbean Public Health Agency. CARPHA Urges Region to Prepare for the Possibility of a Severe Outbreak of Dengue Fever > CARPHA. In: CARPHA [Internet]. 17 Jan 2019 [cited 24 Jan 2019]. http://carpha.org/articles/ID/196/CARPHA-Urges-Region-to-Prepare-for-the-Possibility-of-a-Severe-Outbreak-of-Dengue-Fever

[8] Stanaway JD, Shepard DS, Undurraga EA, Halasa YA, Coffeng LE, Brady OJ, et al The global burden of dengue: an analysis from the Global Burden of Disease Study 2013. The Lancet Infect Dis. 2016; 10.1016/S1473-3099(16)00026-8 26874619 PMC5012511

[9] WHO. Dengue: guidelines for diagnosis, treatment, prevention and control. World Health Organization; 2009. 23762963

[10] Mordecai EA, Cohen JM, Evans MV, Gudapati P, Johnson LR, Lippi CA, et al Detecting the impact of temperature on transmission of Zika, dengue, and chikungunya using mechanistic models. PLoS Negl Trop Dis. 2017;11: e0005568 10.1371/journal.pntd.0005568 28448507 PMC5423694

[11] Lowe R, Gasparrini A, Van Meerbeeck CJ, Lippi CA, Mahon R, Trotman AR, et al Nonlinear and delayed impacts of climate on dengue risk in Barbados: A modelling study. PLoS Med. 2018;15: e1002613 10.1371/journal.pmed.1002613 30016319 PMC6049902

[12] Pontes RJ, Freeman J, Oliveira-Lima JW, Hodgson JC, Spielman A. Vector densities that potentiate dengue outbreaks in a Brazilian city. Am J Trop Med Hyg. 2000;62: 378–383. 10.4269/ajtmh.2000.62.378 11037781

[13] Amarakoon AMD, Chen AA, Rawlins SC, Taylor MA. Dengue epidemics–its association with precipitation and temperature, and its seasonality in some Caribbean countries. West Indian Med J. 2004;53: 60.

[14] Depradine CA, Lovell EH. Climatological variables and the incidence of Dengue fever in Barbados. International Journal of Environmental Health Research Int J Environ Health Res. 2004;14: 429–441. 10.1080/09603120400012868 15545038

[15] Hamilton, I. Analysis of dengue cases in Barbados (2004–2013). Final analysis report V1.3. 4R. 20 March 2014.

[16] Amarakoon D, Chen A, Rawlins S, Chadee DD, Taylor M, Stennett R. Dengue epidemics in the Caribbean-temperature indices to gauge the potential for onset of dengue. Mitig Adapt Strateg Glob Change. 2008;13: 341–357. 10.1007/s11027-007-9114-5

[17] Ortiz PL, Rivero A, Linares Y, Pérez A, Vázquez JR. Spatial models for prediction and early warning of *Aedes aegypti* proliferation from data on climate change and variability in Cuba. MEDICC review. 2015;17: 20–28. 26027583 10.37757/MR2015.V17.N2.6

[18] Barrera R, Amador M, MacKay AJ. Population Dynamics of *Aedes aegypti* and Dengue as Influenced by Weather and Human Behavior in San Juan, Puerto Rico. PLoS Negl Trop Dis. 2011;5: e1378 10.1371/journal.pntd.0001378 22206021 PMC3243685

[19] Moore Chester G., Cline Barnett L., Ruiz-Tiben Ernest, Lee Dwayne, Romney-Joseph Harry, Rivera-Correa Efrain. *Aedes aegypti* in Puerto Rico: Environmental determinants of larval abundance and relation to dengue virus transmission. Am J Trop Med Hyg. 1978;27: 1225–1231. 10.4269/ajtmh.1978.27.1225 727328

[20] Johansson MA, Dominici F, Glass GE. Local and Global Effects of Climate on Dengue Transmission in Puerto Rico. PLoS Negl Trop Dis. 2009;3: e382 10.1371/journal.pntd.0000382 19221592 PMC2637540

[21] Chadee DD, Shivnauth B, Rawlins SC, Chen AA. Climate, mosquito indices and the epidemiology of dengue fever in Trinidad (2002–2004). Ann Trop Med Parasitol. 2007;101: 69–77. 10.1179/136485907X157059 17244411

[22] Gharbi M, Quenel P, Gustave J, Cassadou S, Ruche GL, Girdary L, et al Time series analysis of dengue incidence in Guadeloupe, French West Indies: Forecasting models using climate variables as predictors. BMC Infect. Dis. 2011;11: 166 10.1186/1471-2334-11-166 21658238 PMC3128053

[23] Chen AA, Chadee DD, Rawlins SC. Climate Change Impact on Dengue: The Caribbean Experience: University of the West Indies. Phoenix Printery Ltd, Kingston, Jamaica 2006;

[24] Trotman AR, Mahon R, Shumake-Guillemot J, Lowe R, Stewart-Ibarra AM. Strengthening Climate Services for the Health Sector in the Caribbean. Bulletin of the World Meteorological Organization. 2018;67 https://public.wmo.int/en/resources/bulletin/strengthening-climate-services-health-sector-caribbean

[25] Racloz V, Ramsey R, Tong S, Hu W. Surveillance of Dengue Fever Virus: A Review of Epidemiological Models and Early Warning Systems. PLoS Negl Trop Dis. 2012;6: e1648 10.1371/journal.pntd.0001648 22629476 PMC3358322

[26] WHO/WMO. Improving public health decision-making in a new climate. L. Fernandez-Montoya. JS-G and, editor. 2016.

[27] WMO. Health Exemplar to the User Interface Platform of the Global Framework for Climate Services. Geneva: World Meteorological Organization; 2014.

[28] Ziervogel G, Downing TE. Stakeholder Networks: Improving Seasonal Climate Forecasts. Clim Change. 2004;65: 73–101. 10.1023/B:CLIM.0000037492.18679.9e

[29] Climate Services for Health. In: World Meteorological Organization [Internet]. 9 Jun 2016 [cited 24 Jan 2019]. https://public.wmo.int/en/media/news/climate-services-health

[30] Morss RE, Wilhelmi OV, Downton MW, Gruntfest E. Flood risk, uncertainty, and scientific information for decision making: lessons from an interdisciplinary project. Bull Am Meteorol Soc. 2005;86: 1593.

[31] Soares MB, Alexander M, Dessai S. Sectoral use of climate information in Europe: A synoptic overview. Clim Serv. 2018;9: 5–20.

[32] Handel AS, Ayala EB, Borbor-Cordova MJ, Fessler AG, Finkelstein JL, Espinoza RXR, et al Knowledge, attitudes, and practices regarding dengue infection among public sector healthcare providers in Machala, Ecuador. Trop Dis Travel Med Vaccines. 2016;2: 8 10.1186/s40794-016-0024-y 28883952 PMC5531027

[33] Health Exemplar to the user interface platform of the Global Framework for Climate Services (GFCS) [Internet]. Geneva, Switzerland: World Meteorological Association; 2014. http://www.gfcs-climate.org/sites/default/files/Priority-Areas/Health/GFCS-HEALTH-EXEMPLAR-FINAL-14152_en.pdf

[34] Gould S, Rudolph L. Challenges and Opportunities for Advancing Work on Climate Change and Public Health. Int J Environ Res Public Health. 2015;12: 15649–15672. 10.3390/ijerph121215010 26690194 PMC4690946

[35] Maibach EW, Chadwick A, McBride D, Chuk M, Ebi KL, Balbus J. Climate Change and Local Public Health in the United States: Preparedness, Programs and Perceptions of Local Public Health Department Directors. PLoS ONE. 2008;3: e2838 10.1371/journal.pone.0002838 18665266 PMC2474970

[36] Balbus J, Ebi K, Finzer L, Malina C, Chadwick A, McBride D, et al Are we ready? Preparing for the public health challenges of climate change Environmental Defense Fund. 2008;

[37] Are We Ready? Report 2: Preparing for the Public Health Challenges of Climate Change. In: US Climate and Health Alliance [Internet]. [cited 16 Mar 2017]. http://usclimateandhealthalliance.org/post_resource/are-we-ready-report-2-preparing-for-the-public-health-challenges-of-climate-change/

[38] Paterson JA, Ford JD, Ford LB, Lesnikowski A, Berry P, Henderson J, et al Adaptation to climate change in the Ontario public health sector. BMC Public Health. 2012;12: 452 10.1186/1471-2458-12-452 22712716 PMC3418204

[39] Wei J, Hansen A, Zhang Y, Li H, Liu Q, Sun Y, et al Perception, attitude and behavior in relation to climate change: A survey among CDC health professionals in Shanxi province, China. Environ Res. 2014;134: 301–308. 10.1016/j.envres.2014.08.006 25199970

[40] Macpherson CC, Akpinar-Elci M. Caribbean heat threatens health, well-being and the future of humanity. Public Health Ethics. 2015; phv008. 10.1093/phe/phv008 PMC4498417 26180551

[41] Assessing the Relationship between Human Health and Climate Variability and Change in the Caribbean. St. Lucia: Caribbean Environmental Health Instiute (CEHI); 2014.

[42] Heslop-Thoma C, Bailey W, Amarakoon D, Chen A, Rawlins SC, Chadee DD. Vulnerability to dengue fever in Jamaica Climate change and vulnerability. London: Earthscan Publications Ltd; 2008.

[43] Tompkins AM, Di Giuseppe F. Potential predictability of malaria in Africa using ECMWF monthly and seasonal climate forecasts. J Appl Meteorol Climatol. 2015;54: 521–540.

[44] Wimberly MC, Henebry GM, Liu Y, Senay GB. EPIDEMIA-An EcoHealth Informatics System for integrated forecasting of malaria epidemics. 2014. International Environmental Modelling and Software Society (iEMSs). 7th Intl. Congress on Env. Modelling and Software, San Diego, CA, USA,

[45] Wang M, Liu Y, Wimberly MC. On the Design of EPIDEMIAWeb–A Cloud Based Disease Monitoring System. IEEE; 2018 pp. 0574–0577.

[46] Ahmed S, Francis L, Ricketts RP, Christian T, Polson-Edwards K, Olowokure B. Chikungunya virus outbreak, Dominica, 2014. Emerg Infect Dis. 2015;21: 909 10.3201/eid2105.141813 25898214 PMC4412235

[47] Nsoesie EO, Ricketts RP, Brown HE, Fish D, Durham DP, Mbah MLN, et al Spatial and temporal clustering of chikungunya virus transmission in Dominica. PLoS Negl Trop Dis. 2015;9: e0003977 10.1371/journal.pntd.0003977 26274813 PMC4537218

[48] Ryan SJ, Carlson CJ, Stewart-Ibarra AM, Borbor-Cordova MJ, Romero MM, Cox S-A, et al Outbreak of Zika Virus Infections, Dominica, 2016. Emerg Infect Dis. 2017;23: 1926 10.3201/eid2311.171140 29048289 PMC5652428

[49] Ryan SJ, Lippi CA, Carlson CJ, Stewart-Ibarra AM, Borbor-Cordova MJ, Romero M, et al Zika Virus Outbreak, Barbados, 2015–2016. Am J Trop Med Hyg. 2018;98: 1857 10.4269/ajtmh.17-0978 29637883 PMC6086174

[50] Kumar A, Gittens-St Hilaire M, Nielsen AL. Long-term epidemiological dynamics of dengue in Barbados–one of the English-speaking Caribbean countries. Epidemiol Infect. 2018;146: 1048–1055. 10.1017/S0950268818000900 29655390 PMC9184951

[51] Climate change adaptation to protect human health: Barbados [Internet]. Public Health and Environment Department (PHE) of the World Health Organization; http://www.who.int/globalchange/projects/adaptation/PHE-adaptation-final-Barbados.pdf?ua=1

[52] Verret M, Berry P, Fook TCT, Lal A. Assessment of Climate Change and Health Vulnerability and Adaptation in Dominica. 2016 p. 144. 10.3390/ijerph16010070 PMC6339242 30597870

[53] PAHO WHO | Dengue | Annual Cases Reported of Dengue | PAHO/WHO Data, Maps and Statistics [Internet]. [cited 18 Jul 2017]. http://www.paho.org/hq/index.php?option=com_topics&view=rdmore&cid=6290&Itemid=40734

[54] PAHO. Number of Reported Cases of Chikungunya Fever in the Americas, by Country or Territory. [Internet]. http://www.paho.org/hq/index.php?option=com_topics&view=readall&cid=5927&Itemid=40931&lang=en

[55] Spence B, Emmanuel K. Climate change implications for water resource management in Caribbean tourism. WW Hospitality Tourism Themes. 2009;1: 252–268. 10.1108/17554210910980594

[56] Dominica: The impact of Hurricane Maria—Disaster Profile–January 2018—Dominica. In: ReliefWeb [Internet]. [cited 4 Dec 2018]. https://reliefweb.int/report/dominica/dominica-impact-hurricane-maria-disaster-profile-january-2018

[57] Saldaña J. An introduction to codes and coding The coding manual for qualitative researchers. 2009; 1–31.

[58] Saldaña J. The Coding Manual for Qualitative Researchers. Second Edition edition. Los Angeles: SAGE Publications Ltd; 2012.

[59] Huang C, Vaneckova P, Wang X, FitzGerald G, Guo Y, Tong S. Constraints and barriers to public health adaptation to climate change: a review of the literature. Am J Prev Med. 2011;40: 183–190. 10.1016/j.amepre.2010.10.025 21238867

[60] Herdiana H, Sari JFK, Whittaker M. Intersectoral collaboration for the prevention and control of vector borne diseases to support the implementation of a global strategy: A systematic review. PLoS ONE. 2018;13: e0204659 10.1371/journal.pone.0204659 30303996 PMC6179246

[61] Weaver SC, Charlier C, Vasilakis N, Lecuit M. Zika, Chikungunya, and Other Emerging Vector-Borne Viral Diseases. Ann Rev Med. 2018;69: 395–408. 10.1146/annurev-med-050715-105122 28846489 PMC6343128

[62] Moyes CL, Vontas J, Martins AJ, Ng LC, Koou SY, Dusfour I, et al Contemporary status of insecticide resistance in the major Aedes vectors of arboviruses infecting humans. PLoS Negl Trop Dis. 2017;11: e0005625 10.1371/journal.pntd.0005625 28727779 PMC5518996

[63] Stoddard ST, Morrison AC, Vazquez-Prokopec GM, Soldan VP, Kochel TJ, Kitron U, et al The role of human movement in the transmission of vector-borne pathogens. PLoS Negl Trop Dis. 2009;3: e481 10.1371/journal.pntd.0000481 19621090 PMC2710008

[64] Stoddard ST, Forshey BM, Morrison AC, Paz-Soldan VA, Vazquez-Prokopec GM, Astete H, et al House-to-house human movement drives dengue virus transmission. Proc Natl Acad Sci U S A. 2013;110: 994–999. 10.1073/pnas.1213349110 23277539 PMC3549073

[65] Ryan SJ, Lippi CA, Nightingale R, Hamerlinck G, Borbor-Cordova MJ, Cruz BM, et al Socio-Ecological Factors Associated with Dengue Risk and *Aedes aegypti* Presence in the Galápagos Islands, Ecuador. Int J Environ Res Public Health. 2019;16: 682 10.3390/ijerph16050682 30813558 PMC6427784

[66] Liu N. Insecticide resistance in mosquitoes: impact, mechanisms, and research directions. Ann Rev Entomol. 2015;60: 537–559. 25564745 10.1146/annurev-ento-010814-020828

[67] Heintze C, Garrido MV, Kroeger A. What do community-based dengue control programmes achieve? A systematic review of published evaluations. Trans R Soc Trop Med Hyg. 2007;101: 317–325. 10.1016/j.trstmh.2006.08.007 17084427

[68] Parks W, Lloyd L. Planning social mobilization and communication for dengue fever prevention and control. Geneva: World Health Organization, 2004. WHO/CDS/WMC; 2004.

[69] Stewart Ibarra AM, Luzadis VA, Borbor-Cordova M, Silva M, Ordonez T, Beltran-Ayala E., et al A social-ecological analysis of community perceptions of dengue fever and *Aedes aegypti* in Machala, Ecuador. BMC Public Health. 2014; 1135 10.1186/1471-2458-14-1135 25370883 PMC4240812

[70] So J, Kim S, Cohen H. Message fatigue: Conceptual definition, operationalization, and correlates. Communication Monographs. 2017;84: 5–29. 10.1080/03637751.2016.1250429

[71] Mahon R, Greene C, Cox S-A, Guido Z, Gerlak AK, Petrie J-A, et al Fit for purpose? Transforming National Meteorological and Hydrological Services into National Climate Service Centers. Clim Serv. 2019; 13 10.1016/j.cliser.2019.01.002

[72] WMO. Implementation Plan of the Global Framework for Climate Services. World Meteorological Association; 2014. [Internet]. [cited 30 September 2019].http://www.wmo.int/gfcs/sites/default/files/implementation-plan//GFCS-IMPLEMENTATION-PLAN-FINAL-14211_en.pdf

[73] Street R, Jacob D, Parry M, Runge T, Scott J. A European research and innovation roadmap for climate services. European Commission. 2015; [Internet]. [cited 30 September 2019]. https://ec.europa.eu/programmes/horizon2020/en/news/european-research-and-innovation-roadmap-climate-services

