# Supplementary material for: Co-developing climate services for public health: Stakeholder needs and perceptions for the prevention and control of Aedes-transmitted diseases in the Caribbean
Source: PLoS Negl Trop Dis. 2019 Oct 28;13(10):e0007772. doi: 10.1371/journal.pntd.0007772 (PMC6837543; doi:10.1371/journal.pntd.0007772)
Supplement: S4 Text — (DOCX) [file pntd.0007772.s004.docx]

**S4 Text: National and Regional Policy Opportunities**

*National-level opportunities.* With respect to financial capacity, regional climate stakeholders recommended framing climate services for health as a national development priority, thereby attracting funding from international development agencies. The Caribbean climate and health sectors are beginning to work together to attract the resources needed to increase local capacities to develop climate services for the health sector. A high-level policy goal may enhance the partnership between climate and health government institutions. For example, the Sustainable Development Goals (SDG), and the Paris Climate Agreement are international policies that have common priorities and objectives: good health and wellbeing (Objective 3), climate action (Objective13), and partnership for the goals (Objective 17) [1,2]. The Paris Agreement recognizes the need to strengthen the global response to the threat of climate change and to significantly reduce the risks of climate change (Article 2.1), including the risk to human health [3]. Thus it is critical to develop national policy interactions within the SDGs, to avoid policymakers and public health planners operating in silos [4,5].

The National Development Plan, National Adaptation Plan for Climate Change, and National Disaster Management Plans are policy documents being developed by most countries in the Caribbean. The PAHO has also led recent efforts to develop Health National Adaptation Plans focused on climate resilient health systems for Caribbean SIDS [6]. These documents provide a policy mechanism to establish lines of intersectoral and interdisciplinary work, which can include the development of climate services for health. Climate change adaptation/mitigation measures are part of the Intended National Determined Contributions (INDCs) that nations develop under the Paris Agreement. Many of those measures can generate co-benefits or added value for the health sector; co-benefits are the additional benefits that result when nations act to control climate change and adapt to its impacts [7]. For example, adaptation efforts aimed at improving water management could also reduce the burden of water-borne or vector-borne diseases [8]. However, specific measures need to be identified, monitored and evaluated to be included into the country INDCs to be reported to the United Nations Framework Convention on Climate Change (UNFCCC) [9]. This approach also offers the possibility to access funding from the Green Climate Fund (GCF) in priority sectors such as health, food, water security, and livelihoods of people and communities. For example, the GCF funded Piloting Climate Change Adaptation to Protect Human Health (2009-2016) [10] and recently approved (March 2018) and co-funded a 5 year project, “Water Sector Resilience Nexus for Sustainability in Barbados” [11].

Beyond the climate and health sectors, we identified a complex web of institutional actors who can engage strategically in the development of climate services for health including: a) Water agencies and their climate change and SDG goals related to water supply, and water quality (drinking and wastewater) because of their potential links to vector- and water-borne diseases, b) disaster risk management agencies that deal with hydroclimatic risks that impact vulnerable populations and key infrastructure, c) tourism, to protect visitor health, as well as considering human mobility a critical factor for disease transmission, d) private sector vector control companies, e) community based organizations, and f) academic partners, such as the University of the West Indies. Identifying priorities and gaps in specific information would strengthen the partnership amongst the sectors, making more effective the development of climate services for human health beyond the ministries and offices of public health [12,13].

*Regional-level opportunities.* In its role as the Regional Climate Centre (RCC), the CIMH leads the implementation of the GFCS in the Caribbean. In its thrust to develop sector-specific climate information, the CIMH has pursued an interdisciplinary team approach that leverages the synergies offered by lead technical institutions who are intimately familiar with their national, regional and sectoral contexts, and can consistently invest in the co-production of user-driven climate early warning information [14,15]. The Consortium of Regional Sectoral Early Warning Information Systems across Climate Time scales (EWISACTs) Coordination Partners is an inter-institutional alliance for climate resilience that in its form and function reflects good practice that prioritizes cross-sectoral, interagency models of climate service delivery over those that follow a silo-ed ‘build it and they will come’ approach [16]. As of 2015, the CIMH has actively worked on an emerging, multi-pronged health-climate portfolio in collaboration with national and regional partners such as Ministries of Health, NMHSs, the CARPHA, the PAHO, and other international, interdisciplinary research partners [16]. New and emerging research is being conducted to investigate the linkages between climate and vector borne diseases, heat and health, as well as, Saharan dust and health [16]. Work is also being done in climate and agriculture, which supports health and nutrition.

A clear regional opportunity was the “Third Global Conference on Health and Climate: Special Focus on SIDS,” which was held in Grenada in October 2018. The meeting convened Caribbean Ministers of Health, Ministers of Environment, representatives from UN agencies and other key stakeholders to develop an Action Plan on Health and Climate Change for the Caribbean [17].

The Caribbean Community (CARICOM), a group of 20 Caribbean countries, has mandated the Caribbean Community Climate Change Centre to mainstream climate change adaptation strategies into the sustainable development agendas (UNESCO, 2017). For the Caribbean Region, regional perspectives and considerations are relevant for all the countries [18,19]. Successful projects and tools developed in pilot projects, as done in Barbados [20], can be replicated in similar settings in other countries. A demonstration of the benefits of climate services for arbovirus interventions in one country can be used as a model for other productive sectors (tourism, water supply, disaster risk management), and other countries in the region. Regional institutions should work in cooperation to build technical capacities and resilient communities across the region. This is already happening through the Sectoral EWISACTS portfolio and the work of its multi-institutional Consortium, thereby increasing the expertise and awareness of users and providers. CIMH plans to strengthen their RCC platform for engaging stakeholders to share lessons and promote awareness of climate services based on user-needs for all sectors.

**References**

1. United Nations Framework Convention on Climate Change. Paris Fr. 2015;

2. United Nations. Transforming our world: The 2030 agenda for sustainable development. Resolut Adopt Gen Assem. 2015;

3. Segger M-CC. Advancing the Paris Agreement on Climate Change for Sustainable Development. Camb J Intl Comp L. 2016;5: 202.

4. Huang C, Vaneckova P, Wang X, FitzGerald G, Guo Y, Tong S. Constraints and barriers to public health adaptation to climate change: a review of the literature. Am J Prev Med. 2011;40: 183–190.

5. Nilsson M, Griggs D, Visbeck M. Policy: Map the interactions between Sustainable Development Goals. Nat News. 2016;534: 320. doi:10.1038/534320a

6. PAHO/WHO. Climate change and health in small island developing states: A WHO special initiative in collaboration with UNFCC and the Fijan Presidency of COP-23. SIDS in the Caribbean Region [Internet]. Pan American Health Organization; 2018. Available: https://www.paho.org/hq/index.php?option=com_docman&view=download&category_slug=technical-reports-9862&alias=46262-climate-change-and-health-in-small-island-developing-states-1&Itemid=270&lang=en

7. Gould S, Rudolph L. Challenges and Opportunities for Advancing Work on Climate Change and Public Health. Int J Environ Res Public Health. 2015;12: 15649–15672.

8. World Health Organization. Climate-resilient water safety plans: Managing health risks associated with climate variabilitly and change [Internet]. WHO; 2017. Available: https://www.who.int/water_sanitation_health/publications/climate-resilient-water-safety-plans/en/

9. UNFCC. NCD Registry [Internet]. [cited 30 Sep 2019]. Available: https://www4.unfccc.int/sites/NDCStaging/pages/Party.aspx?party=BRB

10. Global Environment Facility. Project 2553. Piloting Climate Change Adaptation to Protect Human Health [Internet]. [cited 30 Sep 2019]. Available: https://www.thegef.org/project/piloting-climate-change-adaptation-protect-human-health

11. Green Climate Fund. Project FP060: Water Sector Resilience Nexus for Sustainability in Barbados (WSRN S-Barbados) [Internet]. [cited 6 Mar 2019]. Available: https://www.greenclimate.fund/projects/fp060?inheritRedirect=true&redirect=%2Fwhat-we-do%2Fprojects-programmes%3Fp_p_id%3D101_INSTANCE_Hreg2cAkDEHL%26p_p_lifecycle%3D0%26p_p_state%3Dnormal%26p_p_mode%3Dview%26p_p_col_id%3D_118_INSTANCE_4ZRnUzRWpEqO__column-2%26p_p_col_pos%3D1%26p_p_col_count%3D2%26_101_INSTANCE_Hreg2cAkDEHL_delta%3D30%26_101_INSTANCE_Hreg2cAkDEHL_keywords%3D%26_101_INSTANCE_Hreg2cAkDEHL_advancedSearch%3Dfalse%26_101_INSTANCE_Hreg2cAkDEHL_andOperator%3Dtrue%26p_r_p_564233524_resetCur%3Dfalse%26_101_INSTANCE_Hreg2cAkDEHL_cur%3D2

12. McNie E. Delivering Climate Services: Organizational Strategies and Approaches for Producing Useful Climate-Science Information. Weather Clim Soc. 2012; 5 (1) 14-26.

13. Miles EL, Snover AK, Binder LCW, Sarachik ES, Mote PW, Mantua N. An approach to designing a national climate service. Proc Natl Acad Sci U S A. 2006; 103 (52) 19616-19623; https://doi.org/10.1073/pnas.0609090103

14. Mahon R, Greene C, Cox S-A, Guido Z, Gerlak AK, Petrie J-A, et al. Fit for purpose? Transforming National Meteorological and Hydrological Services into National Climate Service Centers. Clim Serv. 2019; 13. doi:https://doi.org/10.1016/j.cliser.2019.01.002

15. CIMH, CARDI, CDEMA, CARHA, CTO, CHTA, et al. Consortium of Regional Sectoral Early Warning Information across Climate Timescales (EWISACTs) Coordination Partners’ Terms of Reference [Internet]. Bridgetown, Barbados; 2016 Aug. Available: https://rcc.cimh.edu.bb/files/2016/10/TOR-Sectoral-EWISACTs-Consortium-and-5-Sectoral-Partners.pdf

16. Trotman AR, Mahon R, Shumake-Guillemot J, Lowe R, Stewart-Ibarra AM. Strengthening Climate Services for the Health Sector in the Caribbean. Bull World Meteorol Organ. 2018;67. Available: https://public.wmo.int/en/resources/bulletin/strengthening-climate-services-health-sector-caribbean

17. PAHO/WHO. Caribbean Ministers of Health meet to approve plan on health system resilience in the face of climate change [Internet]. 16 Oct 2018 [cited 6 Mar 2019]. Available: https://www.paho.org/hq/index.php?option=com_content&view=article&id=14736:caribbean-ministers-of-health-meet-to-approve-plan-on-health-system-resilience-in-the-face-of-climate-change&Itemid=1926&lang=en

18. A Caribbean strategy to cope with climate change | United Nations Educational, Scientific and Cultural Organization [Internet]. [cited 31 Jan 2019]. Available: http://www.unesco.org/new/en/media-services/single-view/news/a_caribbean_strategy_to_cope_with_climate_change/

19. Mahon R, Farrell D, Cox S-A, Trotman A, Van Meerbeeck CJ, Barnwell G. Climate Services and Caribbean Resilience: A Historical Perspective. Soc Econ Stud J Univ West Indies Mona Jam. 2018;67: 239–260.

20. Lowe R, Gasparrini A, Van Meerbeeck CJ, Lippi CA, Mahon R, Trotman AR, et al. Nonlinear and delayed impacts of climate on dengue risk in Barbados: A modelling study. PLoS Med. 2018;15: e1002613.
