# Supplementary material for: Co-developing climate services for public health: Stakeholder needs and perceptions for the prevention and control of Aedes-transmitted diseases in the Caribbean
Source: PLoS Negl Trop Dis. 2019 Oct 28;13(10):e0007772. doi: 10.1371/journal.pntd.0007772 (PMC6837543; doi:10.1371/journal.pntd.0007772)
Supplement: S2 Table — Results from surveys are shown as % (n). (DOCX) [file pntd.0007772.s006.docx]

**S2 Table. Preferred training activities identified by health sector survey respondents.** Results from surveys are shown as % (n).

| **Categories** | **% (n)** |
| --- | --- |
| Technical workshop on how to use climate information, data & models and other tools to predict epidemics) | 78.1% (25) |
| Use of GIS (digital maps) to identify areas at risk of vector borne diseases. | 75.0% (24) |
| How to communicate the effects of climate on health to local communities | 75.0% (24) |
| No response | 6.3% (2) |
