# Supplementary material for: Co-developing climate services for public health: Stakeholder needs and perceptions for the prevention and control of Aedes-transmitted diseases in the Caribbean
Source: PLoS Negl Trop Dis. 2019 Oct 28;13(10):e0007772. doi: 10.1371/journal.pntd.0007772 (PMC6837543; doi:10.1371/journal.pntd.0007772)
Supplement: S4 Table — Results shown as % (n). (DOCX) [file pntd.0007772.s008.docx]

**S4 Table. Preferred way of receiving information from an early warning system that predicts arbovirus epidemics.** Results shown as % (n).

| **Categories** | **% (n)** |
| --- | --- |
| Climate and health bulletins (PDF) by email | 90.6% (29) |
| Online interactive GIS platform | 65.6% (21) |
| Internal meetings within your department | 59.4% (19) |
| Climate and health forums (quarterly) | 34.4% (11) |
| Annual climate-health regional meetings | 25.0% (8) |
| Interactive excel spreadsheets | 25.0% (8) |
